# Statistical evidence for the presence of trajectory in single-cell data

**DOI:** 10.1186/s12859-022-04875-9

**Published:** 2022-08-16

**Authors:** Lovemore Tenha, Mingzhou Song

**Affiliations:** 1 Department of Computer Science, New Mexico State University, Las Cruces, USA; 2 Molecular Biology and Interdisciplinary Life Sciences Graduate Program, New Mexico State University, Las Cruces, USA

**Keywords:** Trajectory inference, Minimum spanning tree, Graph-based statistics, Single-cell sequencing, Developmental biology

## Abstract

**Background:**

Cells progressing from an early state to a developed state give rise to lineages in cell differentiation. Knowledge of these lineages is central to developmental biology. Each biological lineage corresponds to a trajectory in a dynamical system. Emerging single-cell technologies such as single-cell RNA sequencing can capture molecular abundance in diverse cell types in a developing tissue. Many computational methods have been developed to infer trajectories from single-cell data. However, to our knowledge, none of the existing methods address the problem of determining the existence of a trajectory in observed data before attempting trajectory inference.

**Results:**

We introduce a method to identify the existence of a trajectory using three graph-based statistics. A permutation test is utilized to calculate the empirical distribution of the test statistic under the null hypothesis that a trajectory does not exist. Finally, a *p*-value is calculated to quantify the statistical significance for the presence of trajectory in the data.

**Conclusions:**

Our work contributes new statistics to assess the level of uncertainty in trajectory inference to increase the understanding of biological system dynamics.

## Background

Inferring global topological patterns in multivariate data is a central step in understanding dynamical systems such as a living cell. Knowledge of global patterns in the data can be used as a basis for deciding the underlying mechanisms. This is especially relevant to developing biological systems. Dynamical processes such as cell differentiation are revealed in lineages of cells progressing from an early state to a developed state. Single-cell technologies that capture these dynamic processes enable our understanding of global topological patterns that can provide insight into developmental biology. The lineages of cells in a developing tissue are treated as trajectories in dynamical systems where each point represents a cell. Dozens of computational methods have been developed to infer trajectories from single-cell data [1]. Most trajectory inference methods attempt to infer trajectory from a graph representation of the single-cell data. Graph-representation learning [2] obtains a low manifold representation of the dynamics in the single-cell data onto which trajectory inference can then be applied. LISA2 employs *k* nearest neighbors (*k*NN) and builds a spanning tree with user specified root and leaves [3]. STREAM, a trajectory inference method that is applicable on both transcriptomic and epigenetic data employs principal graphs to trajectories [4]. Some methods incorporate additional information other than single-cell gene expression to infer trajectories. CellPath incorporates RNA velocity information and employs *k*NN graph to infer trajectories [5].

However, to our knowledge, none of the existing methods address the problem of determining the existence of a trajectory in observed data before attempting trajectory inference. To address this problem, we introduce graph-based statistics which quantify trajectory existence. Minimum-spanning-tree (MST) based statistics have been successfully used in analyzing global structures from large datasets. Krzewina and Saslaw provide a notable example in astronomy [6], where they introduced MST-based statistics that captured filamentary structures in galaxy data. Although our approach is also MST based, we design different statistics to emphasize the existence of a global trajectory structure. Graph-based statistics have connections to topological data analysis (TDA) [7], which makes use of topological algebra and are thus deterministic in nature. As a result, most of the methods are effectively applicable only to datasets that have clearly defined structures such as the torus. Although the use of statistics have been recently introduced in TDA, the direction is still vastly under-explored. In complementary, our work could help answer the statistical significance question for TDA.

## Results

**Fig. 1:**
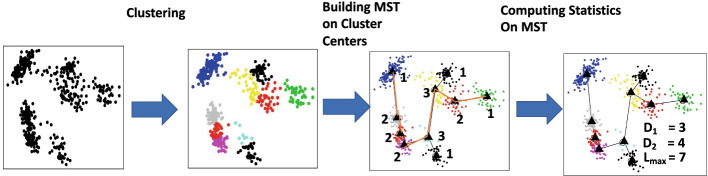
Overview of data transformation steps and trajectory inferential statistics. Characterization of the presence of trajectories is done in a vector space. For example, each point can represent a single cell and the axes expression levels of genes in single cells. The data are first clustered, and an MST is then built on cluster centers. The number next to each node is the degree of the node. The edges highlighted in orange form a longest path in the MST. Three statistics are obtained: (1) $D_1$, the number of leaf nodes is 4, (2) $D_2$, the number of degree-2 nodes is 4, and (3) $L_{\max }$, the length of a longest path in the MST is 7

We present a framework using three graph measures to quantify statistical significance for the presence of trajectory in a given dataset. The framework takes as input a data matrix such as a single-cell RNA-seq dataset, in which each data point is a cell, and the genes are features. For each statistic, the framework gives as output a set of *p*-values, each corresponding to a given number of clusters *k*, such that $a \le k \le b$ where *a* and *b* are minimum and maximum number of clusters, respectively. From the set of *p*-values, the median *p*-value measures the statistical significance for the presence of trajectory in that particular dataset. Figure 1 gives an overview of the framework and illustrates the three test statistics.

Next, we delve into a detailed description of the main steps that the framework undertakes in computing the *p*-values.

For an input multivariate data set *X*, we first perform dimensionality reduction on the data. For each *k* in a given range, a permutation is performed to calculate the distribution of the test statistic under the null hypothesis that there is no trajectory in the dataset. The next step calculates a *p*-value that quantifies the statistical significance for the presence of trajectory in the data.

To capture any global topological structure, the data are first partitioned into *k* homogeneous regions using *k*-means clustering. A weighted undirected graph *G* is created by the *k* cluster centers with edges connecting every pair weighted by their Euclidean distance. An MST *H* is then computed on graph *G* using Prim’s algorithm. We define an MST *H* by a set of *k* nodes $V = \{v_1,v_2,\ldots ,v_k\}$ and $k-1$ edges $E=\{(v_i,v_j)|$ there is an edge between nodes $v_i$ and $v_j$ in *H*, for $i,j = 1,2,\ldots ,k$}. We now introduce three tree-based statistics to characterize the presence of trajectory in the data. These are the number of degree-one nodes $T_1(X) = D_1(H)$, the number of degree-two nodes $T_2(X) = D_2(H)$, and the length of a longest path $T_3(X) = L_{\max }(H)$. Detailed definitions of the statistics and algorithms are given in the “Methods” section.

### Evaluation on simulated and real single-cell data

We demonstrate how the three test statistics report existence of trajectory in single-cell RNA-seq datasets that are simulated or real observations. For single-cell data, the raw dimensionality is typically in the order of tens of thousands. We visualize the data using 2-dimensional principal component analysis (PCA) plots of the datasets. These 2-dimensional data are the input to our analysis. We selected two real datasets, one with trajectory and the other one without trajectory [8–10]. Two simulated datasets were also similarly selected. For each dataset, a permutation test was performed to calculate the empirical distribution of the test statistic under the null hypothesis that there is no trajectory in the dataset. We also selected an additional four simulated datasets to demonstrate the effectiveness of our method in capturing different types of trajectories. The next step involved calculating a *p*-value that quantifies the statistical significance for the presence of trajectory in the data for every value of *k*, the number of clusters, ranging from 5 to 35 clusters. The choice of the range of clusters is critical. Using fewer number of clusters may result in high *p*-values even when there is a clear trajectory in the data. For instance, using three clusters would always result in an MST with the highest degree of linearity. Using too many clusters would also result in high *p*-values even when there is a clearly defined trajectory, since the high number of clusters would result in a highly branched MST. Empirical evidence shows that the number of clusters *k*, such that $5 \le k \le 35$, is reasonable enough to capture significant topological variations in the data. The optimal number of clusters in terms of separability may be too low to capture the statistical significance of the resulting topological structure. Additionally, since our goal is to capture homogeneous regions instead of clustering in terms of separability, we do not attempt to pre-determine the optimal number of clusters to build the MST on. Therefore, the results for each dataset consist of 31 *p*-values corresponding to the number of clusters in the range $5 \le k \le 35$, with each *p*-value quantifying the statistical significance for the presence of trajectory at *k* number of clusters. The median of those *p*-values is then used to represent the overall statistical significance for the presence of trajectory.

**Fig. 2:**
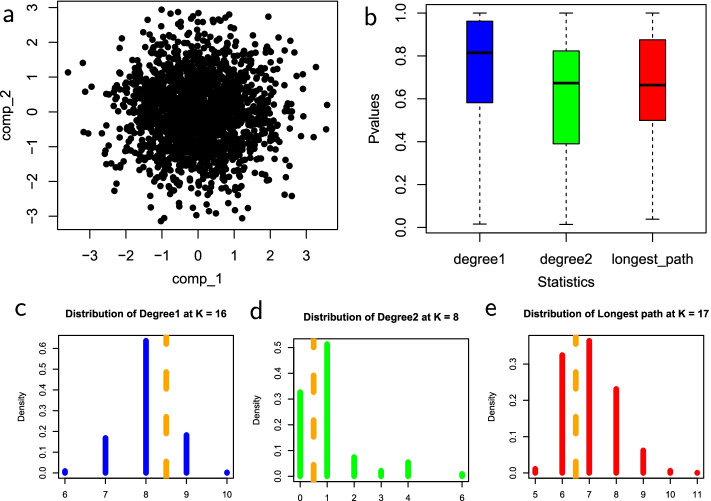
Statistics on simulated single-cell data with no trajectory. **a** Input simulated single-cell data. **b** Box plots of *p*-values obtained for each statistic on the dataset, with the median *p*-values measuring the overall statistical significance for the presence of trajectory. **c**–**e** are null distributions of three statistics obtained by permutation: **c** $D_1$, number of degree-1 nodes, at $k=16$, **d** $D_2$, number degree-2 nodes, at $k=8$, and **e** $L_{\max }$, length of a longest path, at $k=17$. The value of *k* for each statistic corresponds with the median *p*-value of the respective statistic. In each distribution, the observed test statistics are denoted by dashed orange lines

**Fig. 3:**
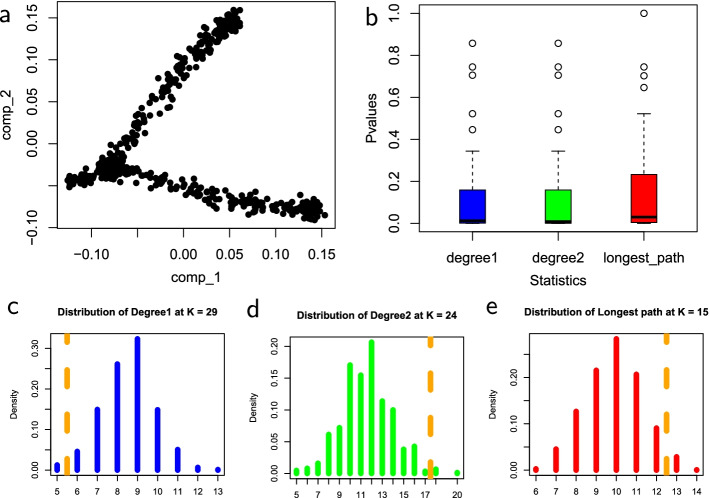
Statistics on simulated single-cell data with a trajectory. **a** Input simulated single-cell data. **b** Box plots of *p*-values for the three test statistics. **c** The null distribution of $D_1$ at $k=29$. **d** The null distribution of $D_2$ at $k=24$. **e** The null distribution of $L_{\max }$ at $k=15$. Figure 2 explains the legend

**Fig. 4:**
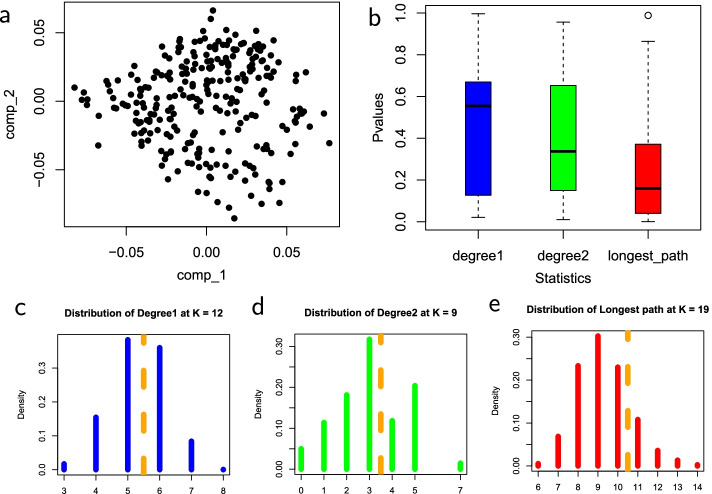
Statistics on noisy single-cell data with a cell-cycle trajectory. **a** Input cell-cycle single-cell data. **b** Box plots of *p*-values for the three test statistics. **c** The null distribution of $D_1$ at $k=12$. **d** The null distribution of $D_2$ at $k=9$. **e** The null distribution of $L_{\max }$ at $k=19$. Figure 2 explains the legend

**Fig. 5:**
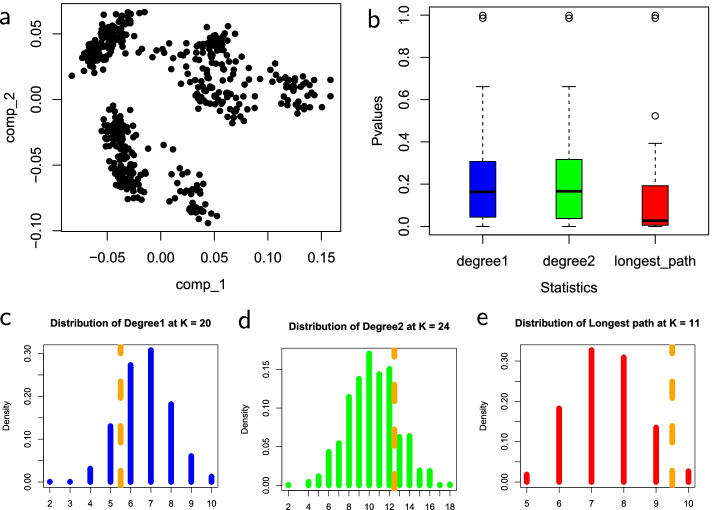
Statistics on mesoderm development single-cell data. **a** Input mesoderm development single-cell data. **b** Box plots of *p*-values for the three test statistics. **c** The null distribution of $D_1$ at $k=20$. **d** The null distribution of $D_2$ at $k=24$. **e** The null distribution of $L_{\max }$ at $k=11$. Figure 2 explains the legend

**Fig. 6:**
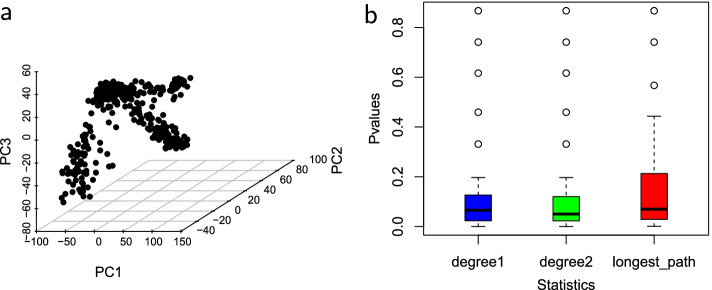
Statistics on fibroblast reprogramming single-cell data. **a** Input fibroblast reprogramming single-cell data. 3-Dimensional scatter plot of the data; points are cells and axes are gene expression. **b** Box plots of *p*-values for the three test statistics

**Fig. 7:**
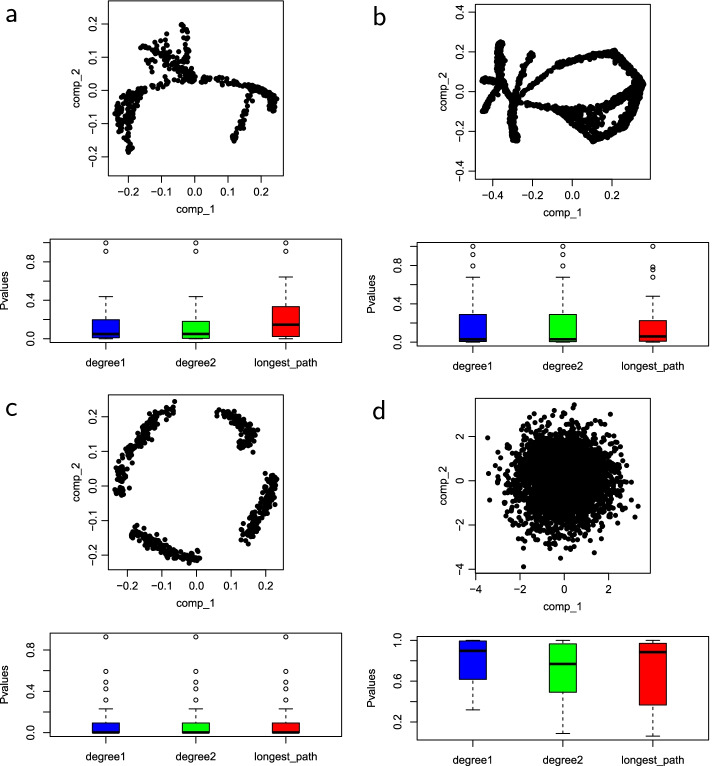
Trajectory presence test statistics on four simulated single-cell data sets with various types of trajectory. For each dataset, three box plots of *p*-values obtained for each test statistic are shown, with the median *p*-values measuring the overall statistical significance for the presence of trajectory. **a** A multifurcating trajectory. **b** A converging-diverging trajectory. **c** A disconnected-looping trajectory. **d** Random data without a trajectory

Figures 2, 3, 4 and 5 show the empirical distributions of the statistics as well as the *p*-values on each of the four datasets. Figure 6 demonstrates the performance of the 3 statistics on a more complex real fibroblast single-cell dataset. Figure 7 shows *p*-values on additional four simulated datasets with different types of trajectories.

The shapes of the distributions of all three test statistics under the null hypothesis that there is no trajectory in the data are roughly similar, with differences in the range and mean values for each statistic. The distributions shown for each statistic were computed at the number of clusters, *k*, that corresponds with the median *p*-value. The *p*-values of the four datasets are consistent with the survey results. Across all three statistics, the median *p*-values, as shown in the box plots, tend to be lower when there is a trajectory in the data, and much higher when the trajectory is not clearly defined. The *p*-values for the number of degree-two nodes statistic tend to be slightly lower in datasets that have no trajectory and slightly higher in datasets that have trajectory. These results demonstrate the effectiveness of these MST-based statistics in reflecting nature and presence of trajectory in datasets.

In Fig. 2, the simulated dataset has no trajectory and the median *p*-values across all statistics are relatively high. The median *p*-values of each statistic are the output of the test: $D_1$ has 0.815, $D_2$ has 0.673 and $L_{\max }$ has 0.664. The results are consistent with the expected output of the method. Since the data have no trajectory, the MST built on the cluster centers would be highly branched resulting in high *p*-values. The dataset provides a good example in which the strength of linearity of the resulting MST is lowest. Since all the statistics can differentiate between extreme cases of linearity, it is no surprise that the *p*-values are relatively high across all statistics. The null distributions of the statistics are relatively comparable, with a unimodal shape that roughly resembles binomial distributions. The main difference in the distributions is the range and mean values of the statistics.

In Fig. 3, the simulated dataset has a clearly defined trajectory. The median *p*-values of the $D_1$ and $D_2$ statistics are 0.013 and 0.008 respectively, which depicts a strong statistical significance for the presence of trajectory. The median *p*-value of the longest-path length statistic is comparably higher at 0.029. Even though the *p*-value is comparably higher, the statistic fairly captures the trajectory. The dataset provides an example in which the degree of linearity of the resulting MST is relatively high. As expected, $D_1$ and $D_2$ statistics best capture the trajectory since they can best detect a high degree of linearity. The shapes of null distributions across all three statistics are also comparable.

In Fig. 4, the dataset is real, single-cell data with a cell-cycle trajectory [11] but also strong noise. The trajectory is not observable on the data presented. The strength of linearity of the resulting MST is relatively low. Therefore, $D_1$ and $D_2$ statistics do not capture the trajectory well as depicted by the median *p*-values. The median *p*-values for $D_1$ and $D_2$ statistics are 0.555 and 0.337, respectively. On the other hand, the median *p*-value of $L_{\max }$ is comparably lower at 0.160. Therefore, the statistical evidence for this true trajectory is low due to strong noise.

In Fig. 5, the dataset is real, mesoderm single-cell data with a development trajectory [9, 10]. The strength of linearity of the resulting MST is high, with few branches. As expected all the statistics capture the trajectory, with relatively low median *p*-values. The median *p*-values of $D_1$ and $D_2$ statistics are 0.163 and 0.167 respectively. $L_{\max }$ has the lowest median *p*-value at 0.027. All the statistics accurately captured the presence of trajectory in the dataset relatively to the noisy cell-cycle data in Fig. 4.

Figure 6 shows the three-dimensional scatter plot and the *p*-values of the three statistics for the trajectory in single-cell data of fibroblast reprogramming. To obtain the data, single-cell RNA sequencing was performed at multiple time points during reprogramming from mouse embryonic fibroblasts to neuronal cells [12]. The median *p*-values of 0.065, 0.05 and 0.069 for $D_1$, $D_2$ and $L_{\max }$, respectively, are relatively significant for all the statistics, demonstrating the effectiveness of the statistics in capturing trajectory presence.

We further demonstrate the effectiveness of the three trajectory presence statistics on four simulated single-cell datasets with three containing trajectories and one containing no trajectory (Fig. 7). Figure 7a shows a multifurcating trajectory. Our method effectively captures this type of trajectory with median *p*-values of 0.048, 0.050 and 0.146 for $D_1$, $D_2$ and $L_{\max }$, respectively, as given in the corresponding box plots. Even though the trajectory has some branches, a large enough number of clusters would capture the linearity of the trajectory. Figure 7b shows a converging-diverging trajectory. The trajectory is captured with median *p*-values of 0.029, 0.029 and 0.060 for $D_1$, $D_2$ and $L_{\max }$ statistics, respectively. Figure 7c shows a disconnected-looping trajectory. The statistical significance for the presence of this trajectory is the most significant, with a median *p*-value of 0.002 for all the 3 statistics. The MST in that dataset is highly linear and resembles a path graph for the majority of *k*, the number of clusters that the MST is captured at. Figure 7d has no trajectory, and as expected, the median *p*-values for all the three statistics are relatively high, at 0.898, 0.769 and 0.884 for $D_1$, $D_2$ and $L_{\max }$, respectively.

## Discussion

### Effectiveness of MST statistics

Our work aims to identify statistics that optimally characterize and distinguish the trajectory structures of data between dynamic patterns and random data. All the three statistics are either maximized or minimized if and only if there is a linear trajectory. The statistics promote trajectory patterns, and non-randomness is between linear pattern and star trees, when there is maximum branching. Empirical studies revealed that the statistics may miss some non-random patterns that are not trajectories such as discrete clusters. There is, therefore, relatively high confidence that most of the non-random patterns captured by the statistics are trajectories.

### Clustering and median *p*-value

We employ clustering to partition the data into homogeneous partitions, which are ideal for capturing trajectory-like structures. Intuitively, different numbers of partitions on the same data may capture distinct types of structures. However, when the trajectory is perfectly linear, different numbers of partitions capture the same underlying trajectory structure. By trying out different numbers of partitions, our empirical studies have demonstrated that the median *p*-value is the best representative and more conservative value to quantify presence of trajectory. In the future, we plan to find a theoretical basis for the choice of the median *p*-value.

Using *k*-means clustering to capture homogeneous regions in the data works but is not ideal. We hypothesize that the accuracy may be improved if the partitioned regions closely resemble each other.

### Null distribution

Computing the empirical distributions of the statistics is CPU intensive due to permutation testing. Our future work will focus on optimizing the computation process as well as identifying analytical null distributions of the test statistics to gain both precision and efficiency.

## Conclusions

We address the problem of statistical evidence for the presence of trajectory on multivariate data. We employed three statistics based on minimum spanning trees to differentiate between data with and without a trajectory. We applied the framework on both real and simulated single-cell RNA-seq datasets and the empirical distributions of the statistics are comparable. The *p*-value measures the statistical significance for the presence of trajectory in a dataset. The effectiveness of the statistics in detecting trajectory existence lays the ground work for further development of efficient algorithms for high dimensional data such as those from single cell biology.

## Methods

The method to obtain the *p*-values for each statistic is summarized in the algorithms below. The main entry point is the Test-Trajectory-Presence algorithm, which takes as input multivariate data and returns a set of *p*-values. The Test-Statistic algorithm calculates the test statistic. The Null-Distribution algorithm obtains the null distribution of the test statistic by permutation.
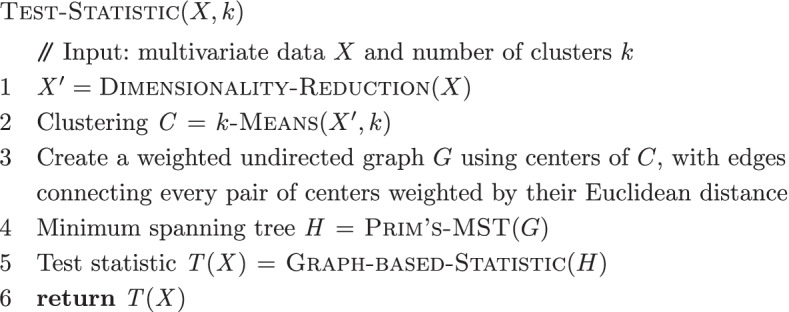

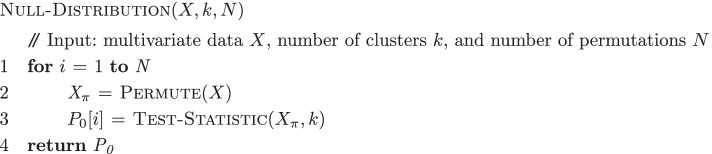

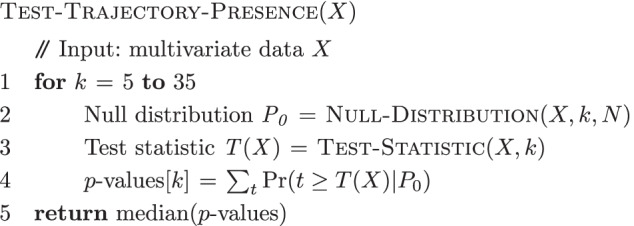


### The number of degree-one nodes or leaves

The first statistic is the number of degree-one nodes or leaves in a tree graph, $D_1(H)$, defined by

1 $$\begin{aligned} D_1(H) = \left| \{v \mid \text {degree}(v) = 1, v \in V\}\right| \end{aligned}$$

The degree of a node in a tree is defined as the number of edges incident on that node. We define a leaf as a node with degree one in a tree. We hypothesize that if there is a trajectory in the data, the MST built on the cluster centers would have fewer branches and thus tends to be more linear. Number of leaves is minimized when the tree is completely linear, when there are no branches in the tree. Such linear trees are also called path graphs. On the other end of the spectrum, the statistic is maximized when the tree is a star tree. In a tree with *k* nodes, the minimum number of leaves is two and the maximum number of leaves is $k-1$. As a result, the number of leaves statistic is quite effective in differentiating between the extremes of linearity of the tree. However, the statistic is not discriminating for some cases in the middle of the spectrum where there might be a noisy global linear trajectory but the number of leaves is not minimized. As MST *H* is derived from the input data *X*, we also call the corresponding test statistic $T_{1(X)}=-D_{1(H)}$.

### The number of degree-two nodes

Another closely related test statistic is the number of degree-two nodes in a tree, $D_{2(H)}$, defined by

2 $$\begin{aligned} D_2(H) = \left| \{v \mid \text {degree}(v) = 2, v \in V\}\right| \end{aligned}$$

Similar to the number of leaves, the statistic attempts to capture the linearity of the tree. When a tree with *k* nodes is linear and has no branches, the number of degree-two nodes is maximized at $k-2$. On the other hand, the statistic is minimized at 0, when the tree is a star tree with maximum branching. The statistic, however, is not theoretically optimal in characterizing tree linearity since there are varying degrees of linearity for which the statistic is minimized at 0. As a result, the statistic is sensitive only in the extreme cases when a trajectory in the data is highly linear or when there is no trajectory at all in the data. On the MST *H*, we also call the corresponding test statistic $T_2(X)=D_2(H)$, as *H* is derived from *X*.

### The length of a longest path

Another test statistic is the length of a longest path in the tree. We define a longest path as a simple path in the tree with the most number of edges. The length of a longest path is defined by the number of edges in the path:

3 $$\begin{aligned} L_{\max }(H) = \underset{\text {path } \pi \text { in } H}{\max } \; \text {length of }\pi \end{aligned}$$

where $\pi \subseteq G$ is a path graph and also a subgraph of *G*. This statistic characterizes the linearity of a tree by capturing tree compactness. A more compact tree tends to have more branches and is representative of data with no trajectory patterns. The statistic is minimized at length two on a star tree; it is maximized at length $k-1$ when the tree is a path graph of *k* nodes. On input data *X* which gives MST *H*, the third test statistic is $T_3(X)=L_{\max }(H)$.

### Permutation test

The input data are permuted to generate a null distribution for each statistic. The *p*-values for all the statistics were calculated using one-tailed tests. We hypothesized that a smaller number of degree-one nodes in the MST implies the presence of strong dynamical patterns, and we therefore, performed a lower tail test to compute the *p*-values for the statistic. Conversely, we performed upper tail tests to compute the *p*-values for the number of degree-two nodes and longest-path length statistics.

## Data Availability

Source code and data to reproduce all results are provided at https://www.cs.nmsu.edu/~joemsong/TrajTest/BMC_source_and_data.zip.

## Abbreviations

MST: Minimum spanning tree
TDA: Topological data analysis
PCA: Principal component analysis
RNA: Ribonucleic acid
